# Effects of a 10-week athletic performance program on match performance variables in male professional football players

**DOI:** 10.3389/fspor.2024.1496895

**Published:** 2025-01-08

**Authors:** Sergio Jiménez Rubio, José L. Estévez Rodríguez, Victor Escamilla Galindo, Sergio L. Jiménez-Sáiz, Juan Del Coso

**Affiliations:** ^1^ Sports Science Research Studies, Rey Juan Carlos University, Fuenlabrada, Madrid, Spain; ^2^ Switzerland National Team Soccer, Muri bei Bern, Switzerland; ^3^ Research Department, ThermoHuman, Madrid, Spain

**Keywords:** elite athlete, conditioning, soccer, mobility, stability, performance

## Abstract

**Introduction:**

The aim of this study was to evaluate the effect of an Athletic Performance Program (APP), implemented as a complement to the usual training routines of a professional football team, on match performance variables in professional football players. The APP was designed to target mobility, stability, strength, multidirectional and sprint skills, which are critical for performance during competitive matches.

**Methods:**

A prospective quasi-experimental study was conducted over three consecutive seasons. Fifty-four professional football players were randomly allocated into a control group (CG, *n* = 25) and an experimental group (EG, *n* = 29). During the in-season period, both groups followed the standard training routines prescribed by the coaching staff. Additionally, the CG performed a general supplementary physical fitness program five times per week, while the EG undertook the APP with the same frequency. The APP included indoor track sessions and micro-doses of on-field stimuli, focusing on specific performance attributes. The interventions lasted 10 weeks. Performance metrics were assessed pre- and post-intervention using GPS to measure match-related variables (total running distance, sprint distance, number of sprints, and peak running speed) and countermovement jump tests to evaluate vertical jumping ability. A two-way ANOVA (2 × 2; group and time) was employed to analyze the effects of the interventions and their interaction.

**Results:**

Significant group × time interaction effects were observed for total running distance (F = 51.853, *P* < 0.001), sprint distance (F = 197.610, *P* < 0.001), number of sprints (F = 86.923, *P* < 0.001), and peak running speed (F = 81.351, *P* < 0.001) during matches. Post-hoc pairwise comparisons revealed that only the EG showed improvements across all performance variables: total running distance (117.5 ± 5.20 to 123.1 ± 3.5 m/min, *P* < 0.001), sprint distance (6.45 ± 1.43 to 8.35 ± 1.22 m/min, *P* < 0.001), number of sprints (0.14 ± 0.03 to 0.16 ± 0.03 sprint/min, *P* < 0.001), and peak running speed (31.1 ± 1.3 to 32.1 ± 1.0 km/h, *P* < 0.001). Conversely, the CG exhibited significant declines in these metrics post-intervention (*P* < 0.001).

**Discussion:**

The findings demonstrate that integrating a 10-week multicomponent Athletic Performance Program into traditional training routines is effective in enhancing match performance variables, particularly in high-intensity actions such as sprints and running speed. The APP's focus on mobility, stability, strength, and sprint-specific skills likely contributed to these improvements, highlighting the importance of targeted supplementary training for optimizing athletic performance in professional football players. The observed decline in the CG emphasizes the need for specialized interventions to maintain and improve performance during the season.

## Introduction

Football is a complex team sport characterized by the repetition of high-intensity actions interspersed with recovery periods of lower exercise intensity. Physical abilities associated with repeated sprinting, accelerating, jumping, and changing direction are essential contributors to the potential performance of football players (1, 2). At the elite level, players may be involved in more than 60 competitive matches (3) throughout a ∼45-week season. This means that professional players are exposed at least to one match per week while there are specific times of the season when multiple matches are played within the same week (4). This demanding calendar compels professional football teams to prioritize recovery in their training schedules, often at the expense of incorporating adequate physical training stimuli throughout the week. In addition, professional football teams devote a considerable amount of training time to develop tactical actions on the pitch to prepare against the characteristics of the next rival, further limiting the time employed to condition players for the physical demands of the game. From a practical standpoint, there might be a need to implement more specific physical training programs to enhance the preparation of professional football players (1, 2) beyond the traditional routines developed on the pitch. These training programs should be focused on the development of players’ physical performance while ensuring an adequate provision of recovery between matches. For this reason, physical training programs performed in addition to team tactics exercises should be individualized and take into account player's involvement in previous matches.

The addition of strength/power training programs to routine football training may favor a more integral physical fitness development of the player (5). The associated improvements in performance parameters (e.g., jump, sprint, COD performance) potentially achieved with the strength training activities may increase a player's ability to cope with training and competition demands (5). The inclusion of training activities to develop other training capacities is also necessary to optimize in-season player's performance as not only strength values are affected by the season. Other variables such as ankle (6) or hip range of motion (7) decrease along the season. Restoring joint range of motion through mobility exercises has previously shown benefits in football players such as increased acceleration and running speed (8–10). The inclusion of training sessions designed to restore joint mobility could be highly beneficial for maintaining players’ performance throughout the season. Recent findings suggest that the traditional 72-hour recovery period, commonly used to separate matches from the first high-intensity training session, may be insufficient for mitigating injury risk factors (11). Collectively, all this information suggests that a multicomponent physical training program performed in addition to normal football training routines may favor several aspects of football performance. The literature supports the implementation of an “extra” program integrated into the standard training regimen of elite football players, with a specific focus on enhancing mobility, stability, and strength development. However, the literature about the efficacy of multicomponent training programs is scarce and no investigation has measured the utility of this type of “extra” training on performance during match play when compared to a control group. Hence, the aim of this study was to evaluate the effect of a customized Athletic Performance Program (APP), consisting of mobility, stability, strength, sprint micro-dosing abilities and multidirectional and linear running skills, and performed in addition to teams’ regular training routines, on match performance variables in professional football players. We hypothesized that the APP implemented by the experimental group would lead to an increase in match performance variables and would be instrumental in reaching the final stretch of the season with improved variables.

## Methods

### Participants

Initially, a total of sixty Spanish male professional football players (age: 26.1 ± 3.4 years, height: 182.3 ± 2.4 cm, body mass: 71.7 ± 2.3 kg, body mass index: 20.8 ± 0.9 kg·m^−2^) voluntarily participated in the study. A *a priori* sample size calculation indicated that at least 16 participants per group were required to obtain statistically significant differences between the control and experimental groups for the changes induced by training on total running distance during a match. The required sample size was calculated to obtain an effect size of 0.25 Cohen's *d* units with a statistical power of 0.890, a two-tailed α level of 0.05, for a two-way ANOVA with within-between interaction. Finally, fifty-four football players were included, with 29 participants in the Experimental Group (EG) and 25 participants in the Control Group (CG). The sample size was calculated using the G*Power software (v.3.1.9.7, Germany). These players could be considered as Tier 4: Elite/International Level according to the classification established by McKay et al. (12). All participants played at the same professional football team competing in the first division of Spanish football (LaLiga). Data was collected for three consecutive seasons (2019–2020; 2020–2021; 2021–2022) with team players who met the inclusion criteria. On average, players trained for 9.52 ± 0.82 h per week and participated in one match per week during the three seasons. The inclusion criteria for the study were as follows: (a) professional football player (>18 years) with no history of cardiovascular or metabolic pathologies and having no injuries during the process of the investigation, (b) no musculoskeletal injury in the month before the onset of the investigation. Players with a history of knee surgery were also excluded from the study. Four players were discarded due to injury within the intervention for causes unrelated to the study and two players decided to drop the experiment. All football players were provided with detailed information about the study procedures, potential risks, and benefits before they signed a written informed consent form. The study was in accordance with the Declaration of Helsinki (2013), and the data collection procedure was approved by the ethical committee of the Universidad Rey Juan Carlos (code: 3105202214522).

### Study design

**Figure 1:**
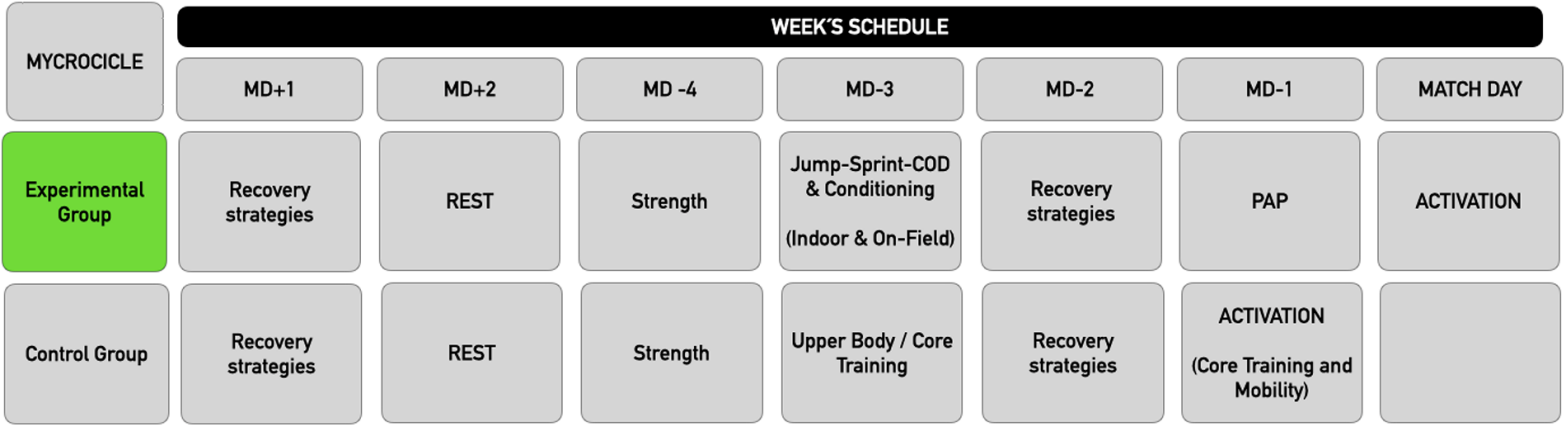
Training microcycle (EG & CG). MD, match day; COD, change of direction; PAP, post-activation potentiation.

A prospective quasi-experimental study was designed to determine the effect of a 10-week Athletic Performance Program (APP) in the experimental group focusing on mobility, stability, strength, sprint micro-dosing abilities and multidirectional and linear running skills on match performance variables in professional football players. Participants in the EG underwent the APP in addition to their normal on-field training routines. Participants in the CG also did an extra physical training program in addition to the field training, with general content on mobility, stability and strength (Figure 1).

Both, the program developed by the EG and the CG were agreed upon and scheduled with the supervision of the team's coaching staff. Before and immediately after the training program, performance variables were measured in official matches to certify the potential effectiveness of the program. Data was collected for three consecutive seasons (2019–2020; 2020–2021; 2021–2022) with team players who met the inclusion criteria. During these three seasons, the team's strength and conditioning staff was the same and data were collected between October and November for the pre-intervention measurement (once the team had played 6–8 official games) and between February and April for the post-training measurement. In that sense, previous studies used similar measurements with athletes (13).

### Athletic performance program (APP)

**Figure 2:**
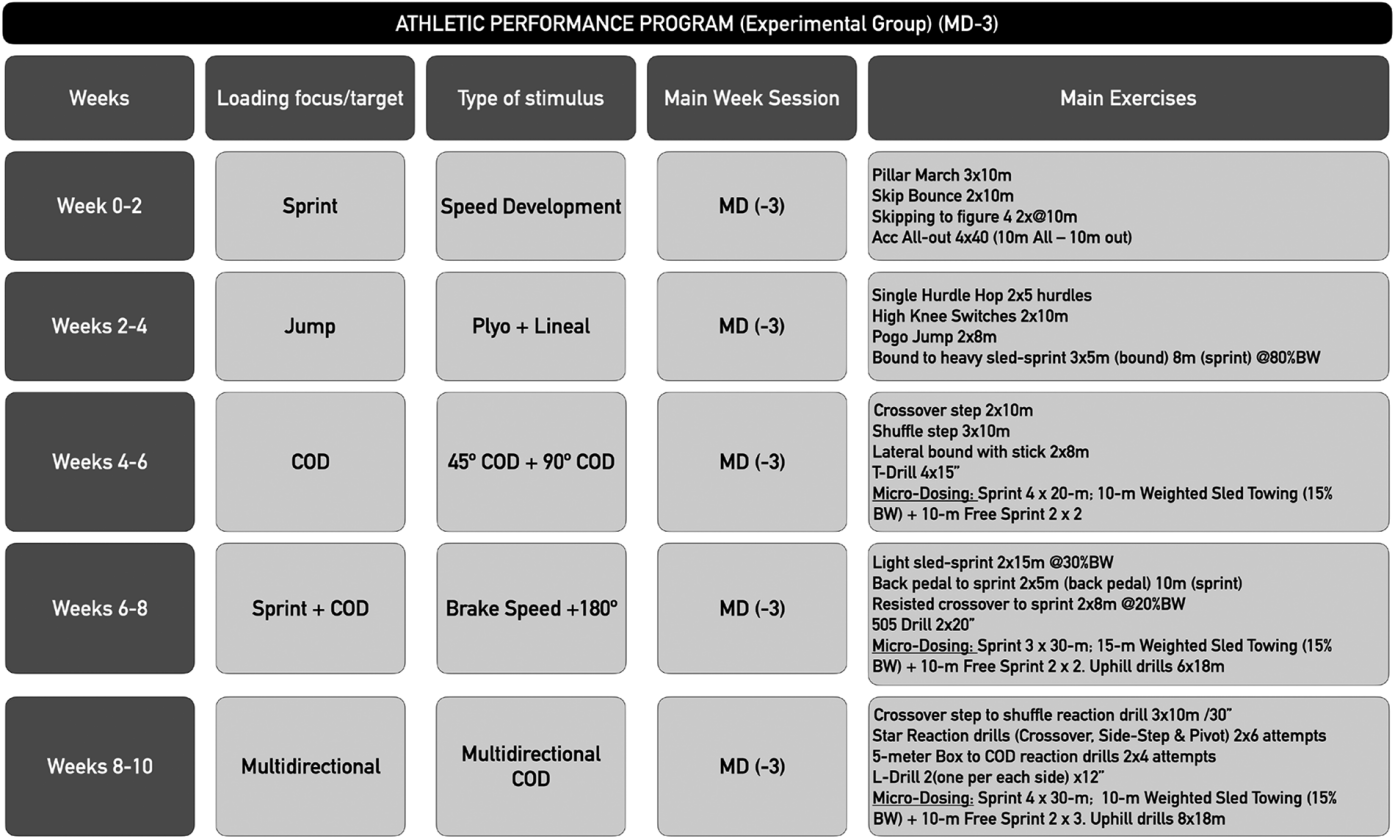
Periodization of the training days focused on linear, multidirectional mechanics, micro-dosing sprint and plyometric training development for a 10-week athletic performance program (EG) in MD-3. MD, match day; COD, change of direction; ACC, Accelerations; Plyo, plyometrics.

The APP implemented in the EG consisted of a set of exercises focused on the development of mobility, stability, strength, sprint ability drills and multidirectional and linear running skills and was predominantly performed indoors with some exercises on-field. The program lasted 10 weeks and included 5 weekly sessions with 30 min per session (14). In each week, the structure was the same taking into account the last competitive match, accounted as match day (MD) The training days of the EG were either focused on recovery and developing mobility and stability (performed on MD +1 and MD +2 and MD −2), on developing lower limbs muscle strength (performed on MD −4) and on enhancing multidirectional, linear running performance and micro-dosing sprint (performed on MD −3) In addition, micro-doses of field training were applied between weeks 4 and 10 of the program, exclusively on match day MD −3 Figure 2). These micro-doses included acceleration-sprints, uphill running and also with other efforts in different sprints of 4 × 20 m (weeks 4–6) and 3–4 × 30 m (weeks 6–10) Between weeks 6 and 10 of the program on MD −3 days the uphill exercise load was 6 × 18 m (weeks 6–8) and 8 × 18 m (weeks 9–10), sled tow at 15% of Body Weight (BW) + 10 m free sprint 2 × 2 plus maximal speed efforts between weeks 4–10 of the program, also on MD −3 days. Other studies in hockey have also applied this micro-dosing concept to assess sprint performance improvement (14).

**Figure 3:**
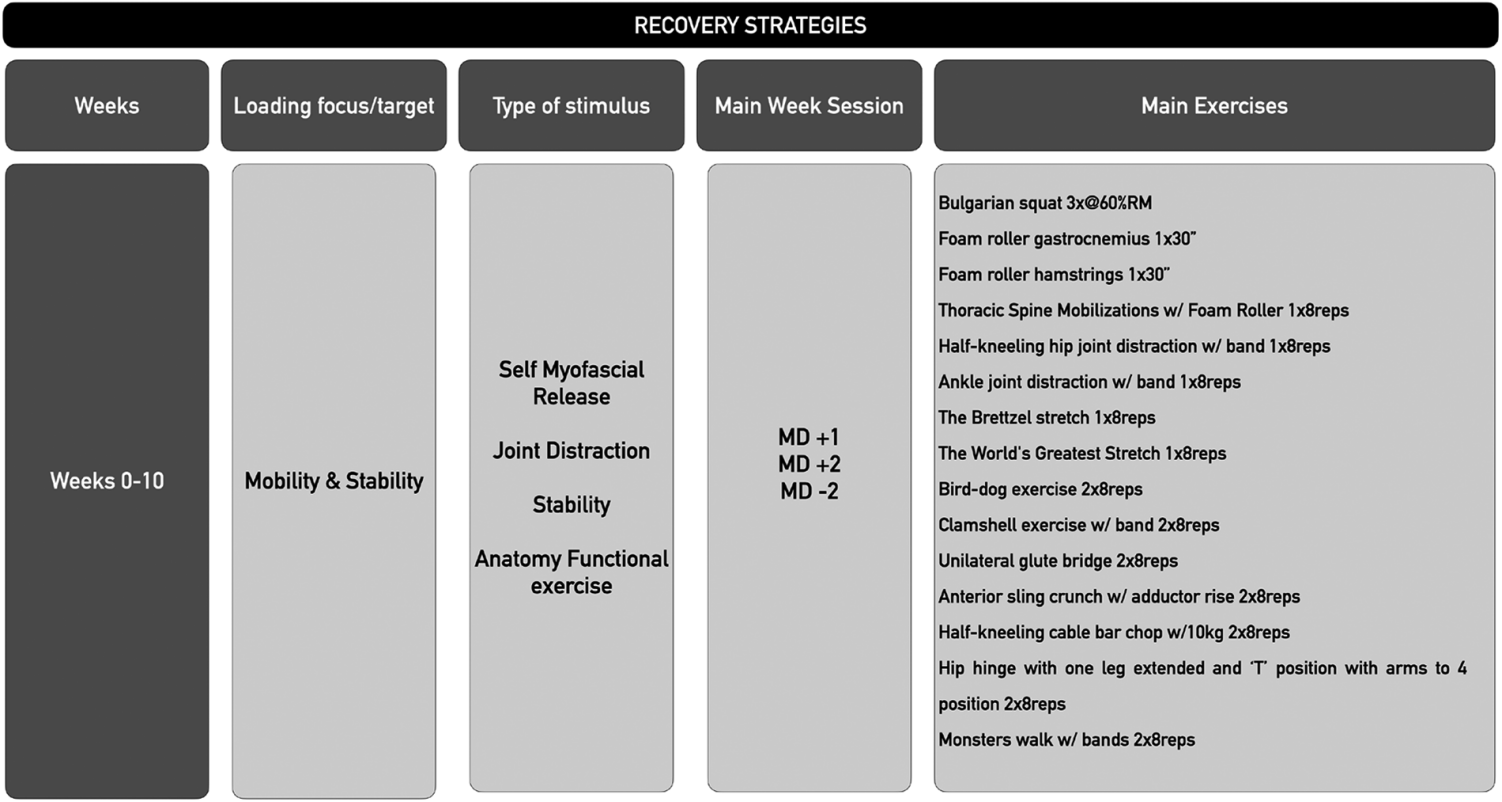
Periodization of the training days focused on mobility and stability tasks for a 10-week athletic performance program. MD, match day.

The APP had a daily undulating periodization (DUP) from the perspective of the weekly microcycle (15). The APP took into account the time match play of each player in the prior competitive match In the weeks with more than one competitive game, only training days with a focus on recovery/mobility/stability were applied for players that completed the games, while a normal training week was performed by those players with less than 30 min of match play (i.e., substitutes). Specifically, the recovery/mobility/stability days were focused on enhancing the ranges of motion (ROM) of the thoracic spine, hip and ankle and achieving optimal development of lumbopelvic control. The main methods used were exercises that included the use of foam rollers and elastic bands for joint distraction (16, 17) (Figure 3). Stability and motor control exercises were also used in the recovery/mobility/stability days to enhance the tolerance of the muscles of the trunk and stabilizers of the pelvis (18–20). Previous studies justify the importance of increasing gluteal activation to improve knee stability (21), and hip muscles exercises to integrate the movement with the lower limbs (22).

**Figure 4:**
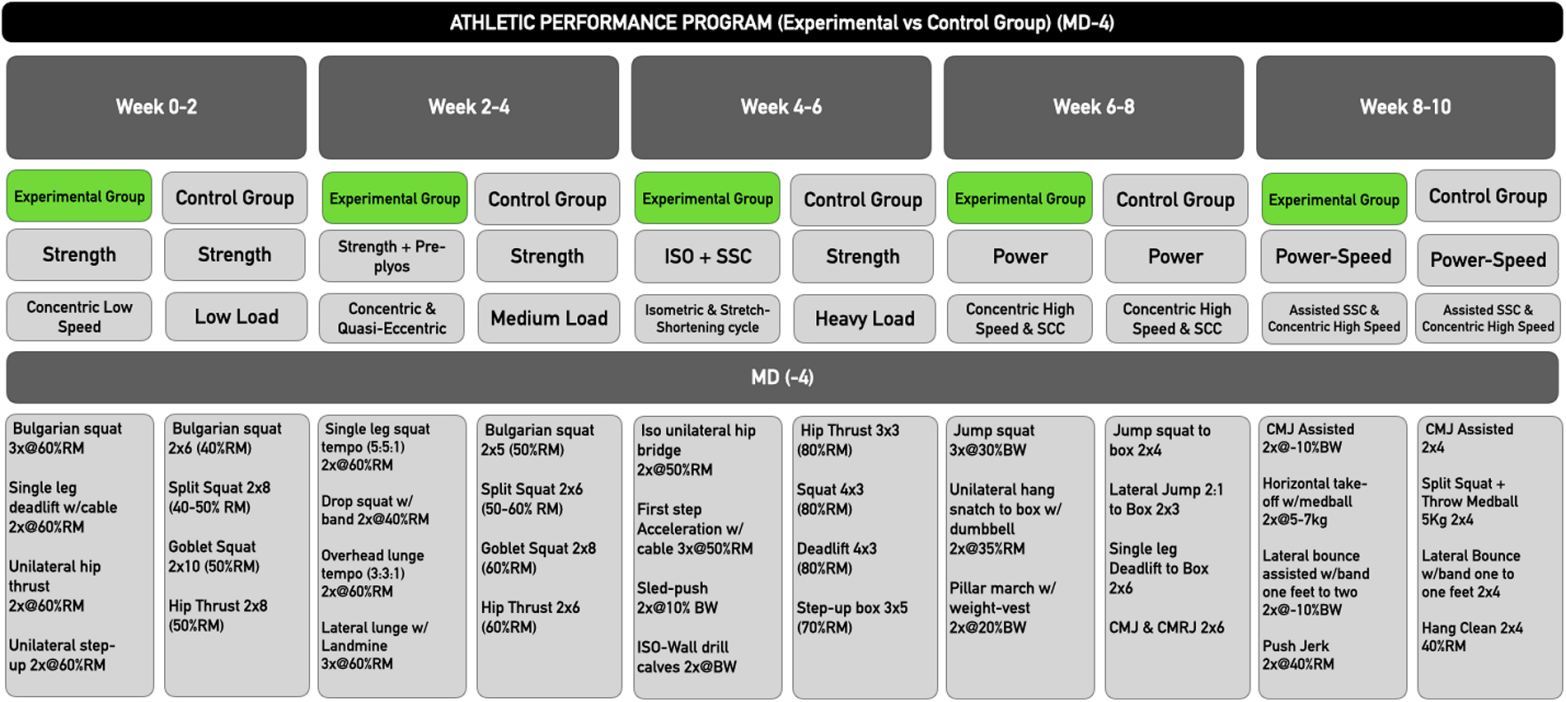
Periodization of the training days focused on strength and power development for a 10-week athletic performance program (EG & GC) in MD-4. MD, match day; ISO, isometry; SSC, stretch-shortening-cycle.

The training days focused on the development of lower limb muscle strength were planned to follow DUP and block periodization (23) (Figure 4). For this purpose, the first 4 weeks had an emphasis on the development of maximal strength with a greater residual effect (24), followed by 2 weeks focused on preparing the tissue for more demanding high-velocity forces that were included in the last 4 weeks, with a power emphasis.

Last, the training days focused on running tasks included exercises to develop linear & multidirectional running mechanics including sprint technical drills and resisted sled training (25) with the intention to improve the application of forces during running (26, 27) as described in the contents of the APP. Moreover, the stimulus with micro-dosing sprint (4 × 20 m/3–4 × 30 m) was applied (Figure 2) (14). On these days, plyometric training was also included to maximize the use of elastic energy and reduce contact time (28), which consisted of applying the maximum possible height and shortest possible contact time through 25 absorptions for session MD−4 in weeks 6–8 and 30 absorptions in weeks 8–10 of the APP.

On the other hand, the Control Group did the same mobility and stability program as the intervention group during MD +1, MD +2 and MD −2. In addition, in MD −4, the CG did traditional lower limb strength work and in MD −3 the extra work was aimed at upper body strength work. However, this program was designed to maintain physical fitness and mobility rather than to promote new physiological adaptations and did not include micro-dosing training or plyometric training.

The external training load was recorded using global positioning systems (GPS) and the internal load was based on a post-training questionnaire on subjective perception of effort, on a scale of 0–10 (CR−10 Borg) (29). The training load was consistently planned and adjusted by the technical and medical staff, while the investigators were responsible solely for monitoring the structure and load of the APP developed for the EG, as well as the equivalent training program implemented for the CG.

### Match performance variables and jump height

#### GPS match variables

Match performance during official matches was recorded by the GPS-accelerometer units (WIMU PRO™; SPRO™, version 989, Realtrack Systems SL, Almería, Spain). The use of these GPS-accelerometer units has been deemed as valid and reliable to assess total distance and distance at different speed thresholds in team sports with coefficients of variations of 2.0% for total running distance and between 4.3 and 15.4% for distance at different thresholds (30). The GPS devices obtained data with a frequency of 18 Hz. According to the manufacturer's recommendations, all the devices were positioned inside a vest and were activated in the middle of the football pitch 15 min before data collection to allow the acquisition of satellite signals and synchronization of the GPS clock with the satellite's atomic clock. Match performance was collected from 4 official matches carried out just before and after the training program. To be considered, the matches should be carried out within the month prior to or after the intervention, and the players had to play at least 80 min in each match. Following each match, data were downloaded to a personal computer and analyzed using a customized software package SPRO^TM^. The variables obtained with the GPS-accelerometer units were: a) Total running distance (m/min); b) High Speed Running Distance (HSRD) which was the distance covered at >21 km/h (m/min); c) accelerations >3 m/s^2^ (number/min); d) decelerations <−3 m/s^2^ (number/min); e) High metabolic load distance (HMLD) which was the distance covered at >25.5 W/kg (m/min); f) peak speed (km/h); g) number of sprints (number/min), taking into account that one sprint was defined as a running action performed at >24 km/h and covering at least 5 m. All variables were normalized by the player's match time (except for peak running speed) as the match duration was not the same in all matches.

#### Vertical jump test

A maximal countermovement jump (CMJ) was performed the week before and the week after the APP, at least 72 h from the prior competitive match. CMJ height was measured with the MyJump 2 App, following the instructions of (31). For the CMJ execution, the player was instructed to rest his hands on his hips while performing a downward movement followed by a maximal effort vertical jump. All players were instructed to reach the maximal height and to land in an upright position, bending knees after landing. Three CMJs were completed after a short warm-up consisting of 5 min of running, 5 mobility exercises and 4 lumbopelvic exercises, and submaximal jumps. There was a 60-second rest between each jump. The mean value of the 3 trials was used for subsequent analyses.

### Statistical analysis

All statistical analyses were carried out in the v28.0.1.9 of the SPSS software (IBM, USA) while the figures have been designed in Excel spreadsheets (Microsoft, USA). Data are presented as mean ± standard deviation (SD) for each intervention group. Initially, the normality of each variable was checked with the Shapiro-Wilk test. Since all variables were normally distributed (*P* > 0.050), parametric tests were selected to examine differences between groups. A two-way analysis of variance [ANOVA; 2 × 2, corresponding to group [APP vs. Control] × time [Pre vs. Post]] was used to compare the effect of each intervention and their interaction. When a significant F value was obtained for any main effect or interaction, a Bonferroni *post hoc* analysis was performed to determine within-group pairwise differences (i.e., pre-to-post training differences) or between group pairwise differences (i.e., differences between groups for the pre- and post-intervention measurements). The effect size was calculated using Cohen's formula (*d*) for paired samples to determine the magnitude of pre- and post-intervention differences within the same group, focusing exclusively on variables that demonstrated statistically significant changes. The magnitude of the effect size was interpreted as follows: trivial (*d* = 0–0.19), small (*d* = 0.20–0.49), medium (*d* = 0.50–0.79) and large (*d* = 0.80 and greater). In all statistical tests, a level of *P* < 0.050 was set to establish statistically significant differences.

## Results

For total running distance during the official matches, there was a main effect of the training group (F = 19.183, *P* < 0.001), a main effect of the time of measurement (F = 8.912, *P* = 0.006) and an interaction between these two effects (F = 51.853, *P* < 0.001). The *post hoc* analysis revealed that the two groups covered a similar running distance before the onset of the interventions (*P* = 0.782). However, only the APP group increased total match running distance in the post-intervention measurement with respect to the pre-intervention measurement (*P* < 0.001, *d* = 1.37, large) while the control group experienced a statistically significant decline after the intervention (*P* = 0.043, *d* = −0.42, small; Table 1).

**Table 1:** Running performance variables during 4 competitive matches and countermovement jump (CMJ) height before (Pre) and after (post) a 10-week athletic performance program (APP) with mobility, stability, strength, and multidirectional and linear running skills or after 10 weeks of routinary training in high-performance football players.

Variable (units)	APP pre	APP post	Δ APP (%)	Control pre	Control post	Δ Control (%)
Total running distance (m/min)	117.5 ± 5.2	123.1 ± 3.5	↑4.9 ± 3.5	117.0 ± 3.3	115.2 ± 4.3	↓−1.5 ± 3.5
Peak running speed (km/h)	31.1 ± 1.3	32.1 ± 1.0	↑3.5 ± 1.9	30.3 ± 1.4	30.1 ± 1.3	↓−0.8 ± 1.2
Number of sprints (n/min)	0.14 ± 0.03	0.16 ± 0.03	↑17.6 ± 14.1	0.13 ± 0.02	0.13 ± 0.02	↓−2.9 ± 5.4
CMJ height (cm)	34.0 ± 2.8	36.0 ± 2.5	↑5.8 ± 2.8	33.5 ± 2.5	33.3 ± 2.4	−0.5 ± 1.4

CMJ, countermovement jump. ↑ Indicates an improvement within the same group, at *P* < 0.050. ↓ Indicates a decline within the same group, at *P* < 0.050.

**Figure 5:**
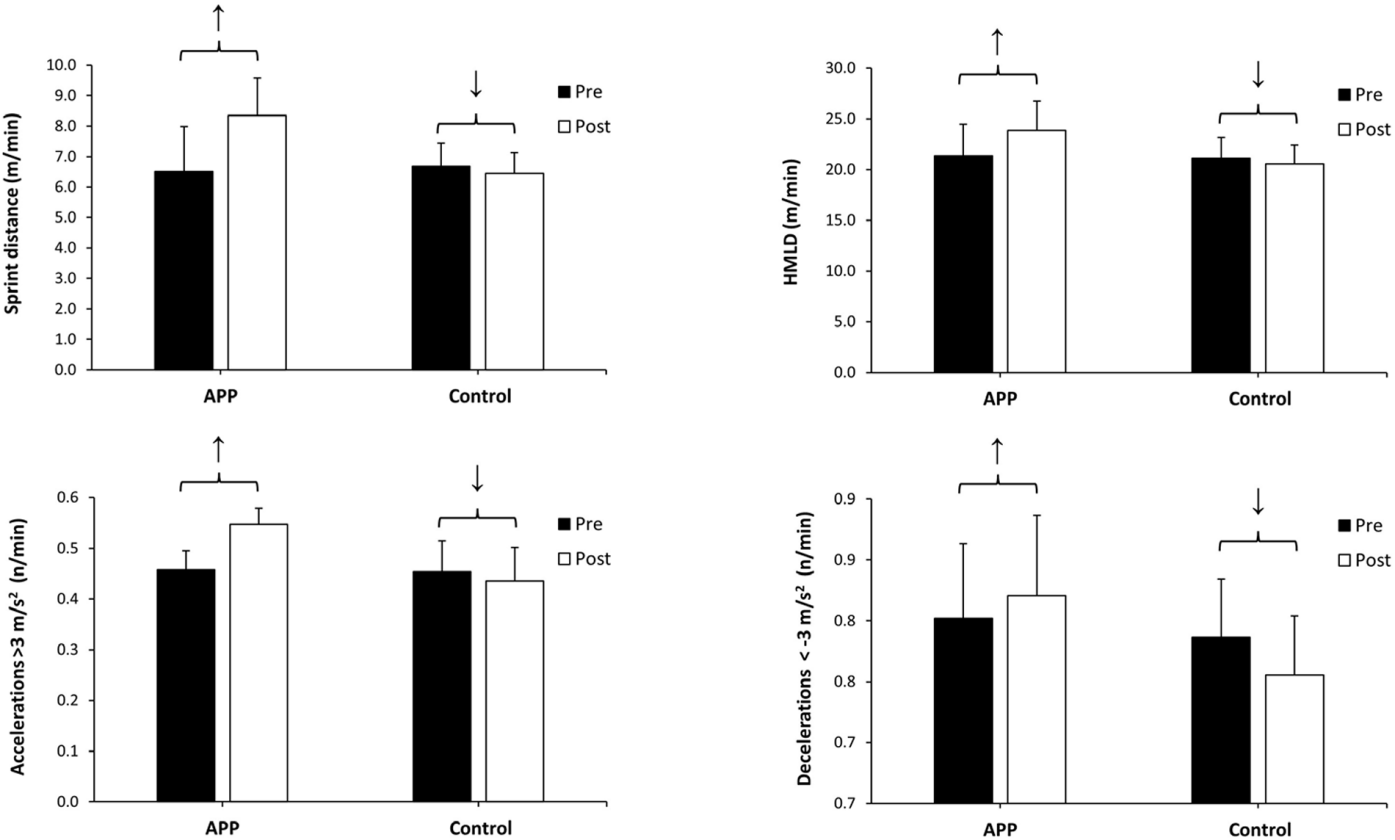
Sprint distance, high metabolic load distance (HMLD) and number of accelerations and decelerations during 4 official competitive matches before (Pre) and after (post) a 10-week athletic performance program (APP) with mobility, stability, strength, and multidirectional and linear running skills or after 10 weeks of routinary training in high-performance football players. ↑ Indicates an improvement within the same group, at *P* < 0.050. ↓ Indicates a decline within the same group, at *P* < 0.050. COD, change of direction; MD, match day; BW, body weigth; Plyo, plyometric; Acc, acceleration.

For the sprint distance covered during the official matches, there was a main effect of the training group (F = 13.701, *P* < 0.001), a main effect of the time of measurement (F = 105.641, *P* < 0.001) and an interaction between these two effects (F = 197.610, *P* < 0.001). The *post hoc* analysis revealed that the two groups covered a similar sprint running distance during the matches performed before the interventions (*P* = 0.734). However, only the APP group obtained higher values of sprint distance in the post-intervention measurement than in the pre-intervention measurement (↑31.2 ± 15.9%, *P* < 0.001, *d* = 1.95, large) while the control group experienced a statistically significant decline (↓3.4 ± 2.5%, *P* < 0.001, *d* = −0.35, small; Figure 5).

For HMLD, there was a main effect of the training group (F = 31.830, *P* < 0.001), a main effect of the time of measurement (F = 55.068, *P* < 0.001) and an interaction between these two effects (F = 125.232, *P* < 0.001). The *post hoc* analysis revealed that the two groups had similar pre-intervention HMLD values during official matches (*P* = 0.162). However, only the APP group obtained higher values of HMLD in the post-intervention measurement (↑12.5 ± 7.1%, *P* < 0.001, *d* = 1.74, large) while the control group experienced a statistically significant decline after the intervention when compared to the pre-testing data (↓2.6 ± 2.3%, *P* < 0.001, *d* = −0.43, small; Figure 5).

For the number of accelerations performed during the official matches, there was a main effect of the training group (F = 33.418, *P* < 0.001), a main effect of the time of measurement (F = 88.650, *P* < 0.001) and an interaction between these two effects (F = 351.169, *P* < 0.001). The *post hoc* analysis revealed that the two intervention groups performed a similar number of match accelerations (*P* = 0.457). The APP group obtained a higher number of accelerations in the post-intervention measurement than in the pre-intervention measurement (↑20.1 ± 8.7%, *P* < 0.001, *d* = 2.30, large) while the control group experienced a statistically significant reduction of the number of accelerations per match (↓3.9 ± 2.3%, *P* < 0.001, *d* = −0.60, medium; Figure 5).

For the number of decelerations, there was a main effect of the training group (F = 7.226, *P* = 0.013), and a group × time interaction (F = 48.095, *P* < 0.001). The *post hoc* analysis revealed that the two intervention groups performed a similar number of match decelerations before the interventions (*P* = 0.291). The APP group obtained higher values of sprint distance in the post-intervention measurement than in the pre-intervention measurement (↑2.3 ± 3.8%, *P* = 0.013, *d* = 0.60, medium) while the control group experienced a statistically significant decline in the number of decelerations (↓3.9 ± 2.2%, *P* < 0.001, *d* = - 0.61, medium; Figure 5).

Regarding peak running speed obtained during the matches, there was a main effect of the training group (F = 30.762, *P* = 0.013), a main effect of the time of measurement (F = 41.764, *P* < 0.001) and an interaction between these two effects (F = 81.351, *P* < 0.001). The *post hoc* analysis revealed even before the onset of the interventions, the players of the APP group had higher values of peak running speed (*P* = 0.012). However, despite this pre-intervention difference, only the APP group improved peak running speed (*P* < 0.001, *d* = 1.82, large) while the control group experienced a slight but statistically significant decline (*P* = 0.020, *d* = - 0.23, small; Table 1).

Regarding the number of sprints during the matches, there was a main effect of the training group (F = 6.934, *P* = 0.015), a main effect of the time of measurement (F = 20.061, *P* < 0.001) and an interaction between these two effects (F = 86.923, *P* < 0.001). The *post hoc* analysis revealed both groups performed a comparable number of sprints before the onset of the interventions (*P* = 0.498). Only the APP group improved the number of sprints per match (*P* < 0.001, *d* = 1.25, large) while the control group completed a lower number of sprints after the intervention (*P* = 0.005, *d* = - 0.58, medium; Table 1).

Last, there was a main effect of the training group (F = 21.792, *P* < 0.001), a main effect of the time of measurement (F = 65.043, *P* < 0.001) and an interaction between these two effects (F = 137.436, *P* < 0.001) in CMJ height. The *post hoc* analysis revealed both groups had similar CMJ height before the interventions (*P* = 0.115) while only the APP group improved CMJ performance with the intervention (*P* < 0.001, *d* = 2.07, large) while the control group experienced no pre-to-post intervention changes (*P* = 0.005; *d* = - 0.36, small; Table 1).

## Discussion

In professional football, maintaining players’ physical condition throughout the season is one of the main aims of the strength and conditioning staff during the competitive period (32). This is due to the demanding competitive schedule of professional football, which involves multiple competitions and at least one match per week. As a result, matches serve as the primary training stimulus for most elite football players during the week. In this context, the training week is developed to recover from the previous match and to condition players for the following match, with very limited time devoted to enhancing physical performance. Additionally, the coaching staff must conjugate multiple objectives for the training practices available such as the preparation of the strategy, the improvement of individual technical-tactical skills, and psychological preparation (32, 33) which further limits the time available for performance development. Collectively, these characteristics of professional football frequently impede the existence of a proper conditioning program during the season (1, 2). In this context, the aim of this study was to evaluate the effect of a customized APP, in addition to regular training routines, on match performance variables of professional football players, comparing those in the intervention group to their teammates in the control group from the same team.

The study was developed within a professional football team to increase the ecological validity of the study outcomes. Overall, the APP of the intervention group increased players’ peak running speed, the number of sprints, and the ability to accumulate sprint distance during matches which constitutes remarkable proof of efficacy for this training program in comparison to the control group of players. These outcomes suggest the utility of including physical preparation programs as an addition to traditional on-pitch football training practices to enhance players’ match running performance at high intensity. In this context, strength and conditioning coaches may consider the use of mobility/stability, strength exercises and running tasks to complement players’ physical condition, particularly in professional football teams with difficulties in reaching a proper training stimulus due to the demands of the calendar. In comparison to the assessment performed before the intervention, the 10-week APP improved high-intensity running performance (i.e., peak speed, sprint distance, number of sprints and HMLD -m/min- and total distance covered) during competitive matches and has a positive influence on mechanical variables such as the number of accelerations and decelerations. The improvements in these variables were statistically significant and meaningful in terms of sports performance as the magnitude of the improvements ranged from 2% to 31% (large effect in all cases according to Cohen's *d* categorization). The wide range and the magnitude of the effects induced by the APP were likely produced by the characteristics of the program, which it included 5 × 30-min sessions per week with training days focused on mobility/stability, strength and running performance through micro-doses of 20–30 m sprint training, as well as uphill actions, especially in weeks 6–10 of the program. Meanwhile, the control group continued with the general routines imposed by the coaching staff and did not follow any performance program, which could be detrimental to players’ capacities. This may be due to the fact that, as literature has shown, the performance of football players is diminished by the competitive schedule, causing players’ performance to experience a decline from the mid-season phase towards the end of the season (34). Interestingly, other specific programs applied to professional football players with 1 session/week of strength training for 12 weeks were effective in maintaining strength, sprint, and jump performance achieved during a preceding 10-week preparatory period but did not report enhancements on these variables. Additionally, previous studies (35) observed that professional players who performed two HIIT sessions in addition to in-season training increased maximal aerobic speed while reducing the time to complete a 40-m sprint. Taken together, all this information suggests that at least two fitness sessions, especially with the concept of extra micro-doses added to the team's regular training routines, are needed to produce improvements in key physical parameters and thereby bring the external load values closer to the actual ones in terms of game demands (Figure 5).

The current investigation is innovative because included a multicomponent training program and because the efficacy of the program has been measured during competitive matches. The improvements in the current investigation provided by the APP program could be attributed to a multifactorial nature (36). The training days devoted to recovery/mobility and stability were more oriented to restoring player's physical capacity after the prior competitive match, but it is likely that these days also contributed, in an indirect manner, to the improved running performance during the matches, through a better range of movement at different joints (37–39) Strength and power exercises were included due to the ample evidence that certifies their usefulness in improving several aspects of sports performance (40). Although both groups engaged in strength training, it is important to note that the EG followed a more specific and innovative program, characterized by clear periodization and progressive loading. In contrast, the CG adhered to traditional strength training aimed primarily at maintaining physical fitness throughout the season. In addition, about the benefits of improving lower body strength and its relationship to fatigue expression and injury prevention, there is limited information. Other authors (41) observed a negative relationship between lower body strength and fatigue markers evaluated through the assessment of muscle damage after a match in football players. Specifically, these authors found that blood creatine kinase (CK) concentration was lower in those players with greater lower body strength, suggesting a preventive role of the development of lower body muscle strength against muscle damage during a competitive football match.

On the other hand, previous studies (34), show that maximum oxygen consumption and the ability to perform intermittent efforts suffer a decline from mid-season to the final stages of the season in professional football players. A recent meta-analysis (42) has justified that high-intensity intermittent and sprint training cause improvements at a physiological level, increasing VO_2_ max as well as improving the capacity to repeat sprints (RSA), one of the determining aspects in the evaluation of performance in football. The inclusion of this type of training in the APP, along with sled training, linear and multidirectional skills training developed at a high intensity (25, 43) could be the main reason for the enhancement of running parameters during the match, in addition to improving the application of horizontal force in decisive actions, such as sprinting (26, 44).

Similarly, the program incorporated plyometric exercises with vertical and horizontal vectors performed at high speeds, which likely contributed to the improved acceleration/deceleration profile of players during matches and enhanced jump performance. These exercises are known to have a significant impact on force development and absorption characteristics during short, intense actions, potentially influencing the adaptive response to training (45). This type of training is expected to have a positive biomechanical impact by promoting more effective and efficient movement patterns, including improved changes of direction as well as enhanced acceleration and deceleration mechanics (46).

After the APP, participants improved running distances at high speed and at high metabolic load, together with an enhanced capacity to accelerate and decelerate and improved jump performance. The obtaining of such improvements was likely propitiated by the type of exercises and the progression of intensity, implemented by 5 × 2 weeks block for the 10-week duration of the APP. Although the present study does not allow us to know which type of training improved running performance during the match, the current study reveals that a multi-component training program can produce significant improvements in football-specific physical capacities. This situation did not occur in the control group, as they followed a general program oriented towards maintaining physical fitness with a general focus. The program followed by the CG shows similarities to the EG, but they did not follow a specific training plan, for the MD−4 and MD−3 days, where the differences between the two programmes seem to be decisive.From a practical standpoint, the use of a physical training program that included several types of exercise -developed during different days- may be more effective than the use of a single-component type of training (e.g., high-intensity intermittent training) as the characteristics of football patterns require players not only to run at high speed but to accelerate in short spaces without the obtaining of high speeds and constant changes of direction. In fact, as some authors point out, each player needs an individualized performance program in order to optimize physical capabilities, with the aim of prioritizing individual demands and taking into account individual external and internal loads (47).

The current study possesses some limitations that should be discussed to contextualize the application of the study. First, as it was a multi-component program, it was not possible to determine which variable was more determinant in performance. Second, pre and post-intervention measurements were done at different moments of the season. Previous data indicate that player's match performance is maintained constant through the season once players have completed the first 6–8 matches (48). For this reason, we included the pre-training measurement in October, after players had played 6–8 competitive matches. In this context, it is likely that the players in the intervention group have achieved their stable match performance. With this information, we sincerely believe that the improvements obtained in this study in match performance variables are the result of the APP and do not reflect changes in performance during the season. Third, this study was performed in a professional football team with a complex competitive calendar. It is possible that other football teams, especially those of lower categories or football level, do not need to implement APP as a less congested calendar allows the development of training week with more time to develop players’ physical conditioning. Finally, we did not take into account the anthropometric evolution of the players and its relationship with the development of performance throughout the season. Despite these limitations, we honestly believe that the study outcomes may be of great utility for strength and conditioning coaches of professional teams with complex competitive calendars.

## Conclusions

The inclusion of a 10-week multi-component training program focused on the development of mobility, stability, strength, sprinting ability, and multidirectional and linear running and sprint skills in the usual training program was effective in the participants who executed it to improve intense actions during play in official matches in professional football players. These outcomes suggest that the addition of a customized andphysical training program to the traditional training week of professional football teams may be an effective strategy to improve players’ high-intensity running during competitive matches. Coaches and strength conditioning staff are continuously looking for the best training approach to optimize professional football players’ performance within the context of the congested calendar that intrinsically accompanies professional football. For this reason, the staff of professional football teams may consider the use of multicomponent training program as an addition to traditional pitch football training to enhance players’ match running performance at high intensity.

### Practical implications

- The biweekly progression of the extra program implemented by the experimental group for mobility, strength and movement and sprint skills is one of the most interesting practical applications derived from the results of this research, which can be taken up by physical trainers in the future to optimize external loading variables that are closer to the real demands of competition.
- Following the APP program, participants in the experimental group improved their running distances at high speed and high metabolic load, along with an increased ability to accelerate and decelerate and improved jumping performance. All parameters are considered relevant for optimizing performance.
- Another important application, is the justification of managing the loads in the microcycle in the load-recovery sequence to understand the load profile and the specific days on which to apply them for future extra programs with elite football players.

## Data Availability

The original contributions presented in the study are included in the article/Supplementary Material, further inquiries can be directed to the corresponding author.
